# Supplementary material for: Characterization of the nuclear and cytosolic transcriptomes in human brain tissue reveals new insights into the subcellular distribution of RNA transcripts
Source: Sci Rep. 2021 Feb 18;11:4076. doi: 10.1038/s41598-021-83541-1 (PMC7893067; doi:10.1038/s41598-021-83541-1)

Supplementary Figure 2

Analysis of transcript distribution between cytosol and nucleus. Shown are the number of transcripts significantly enriched in either cytosol or nucleus in the tissue samples we used in our study and in PolyA+ and polyA- RNA-seq data from the cytosol and nucleus of the ENCODE cell lines.

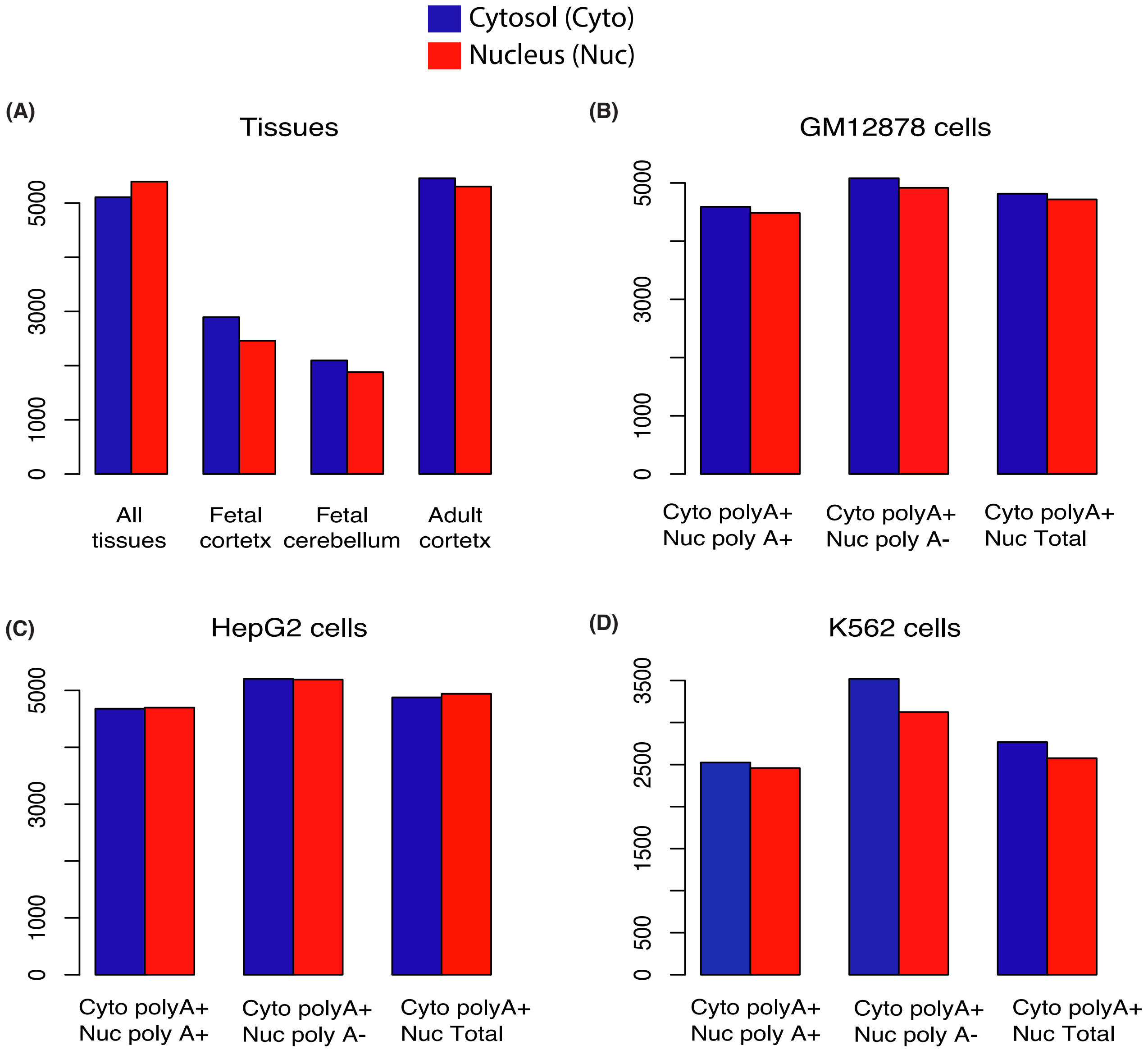

Supplement: Supplementary file 3 — Supplementary Figure S2. [file 41598_2021_83541_MOESM3_ESM.pdf]
