# Supplementary material for: Characterization of the nuclear and cytosolic transcriptomes in human brain tissue reveals new insights into the subcellular distribution of RNA transcripts
Source: Sci Rep. 2021 Feb 18;11:4076. doi: 10.1038/s41598-021-83541-1 (PMC7893067; doi:10.1038/s41598-021-83541-1)

Supplementary Figure 3

A) Mean log2FC in each tissue, for genes divided into groups based on gene length by 10 Kb. Numbers by each bar represents number of genes with positive/negative fold change in each group. **B)** log2FC of fetal cerebellum (blue), fetal frontal cortex (green), and adult frontal cortex (red), lines represent mean fold-change (log2FC > 0, cytosolic; log2FC < 0, nuclear) for genes binned according to transcript length. **C)** Expression using RPKMs of same tissues as in B, lines represent mean RPKM across all samples for genes binned according to gene length.

(A)

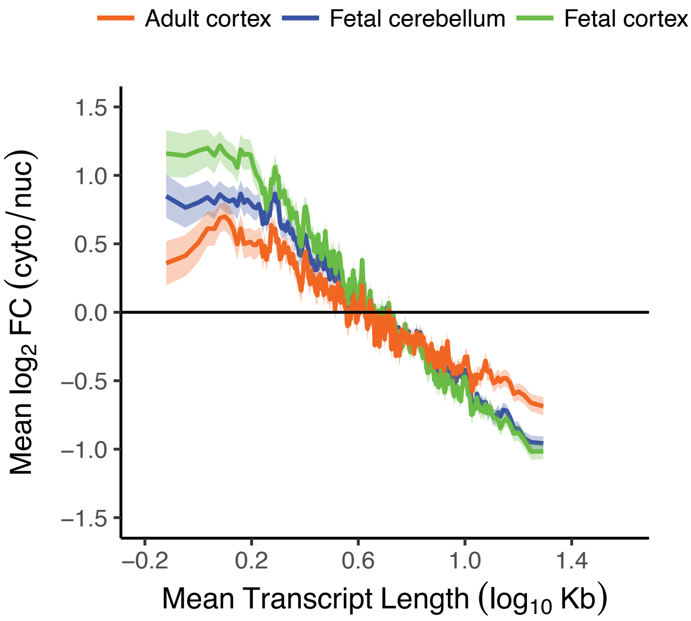

(B)

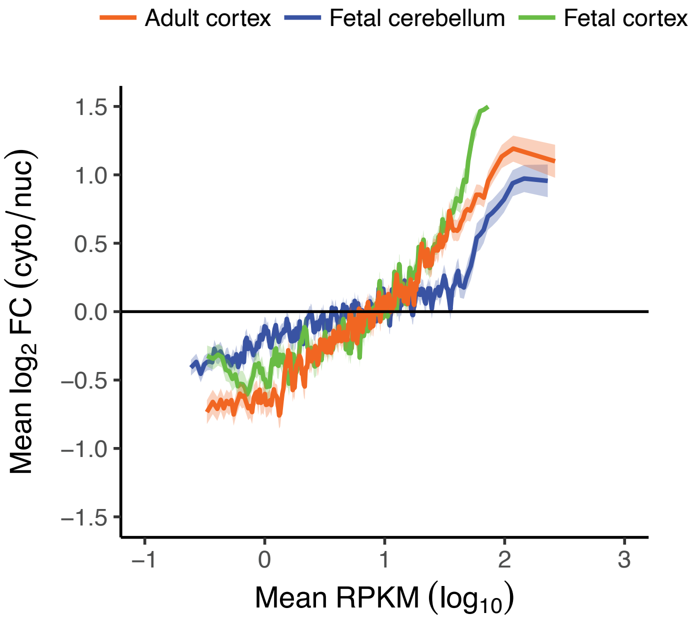

(C)

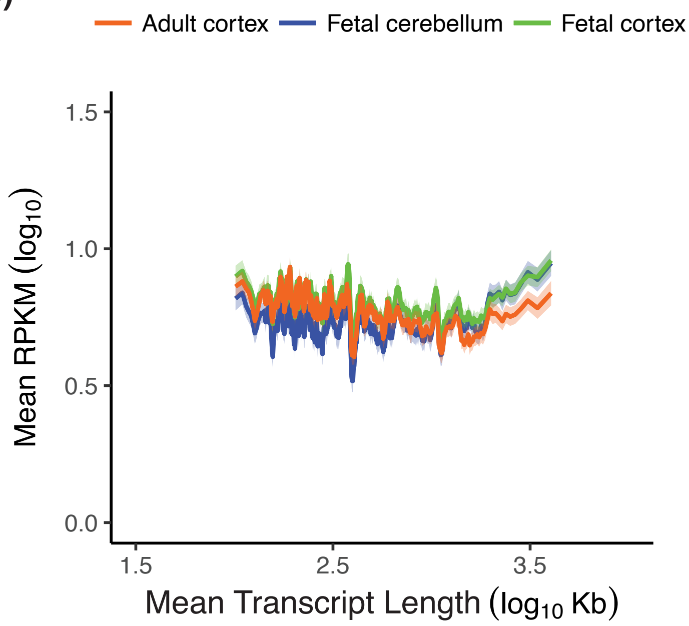

Supplement: Supplementary file 4 — Supplementary Figure S3. [file 41598_2021_83541_MOESM4_ESM.pdf]
