# Supplementary figures and images for: Characterization of the nuclear and cytosolic transcriptomes in human brain tissue reveals new insights into the subcellular distribution of RNA transcripts
Source: Sci Rep. 2021 Feb 18;11:4076. doi: 10.1038/s41598-021-83541-1 (PMC7893067; doi:10.1038/s41598-021-83541-1)

**Supplementary Figure 4**

MA plots and violin plots for each tissue analyzed separately.

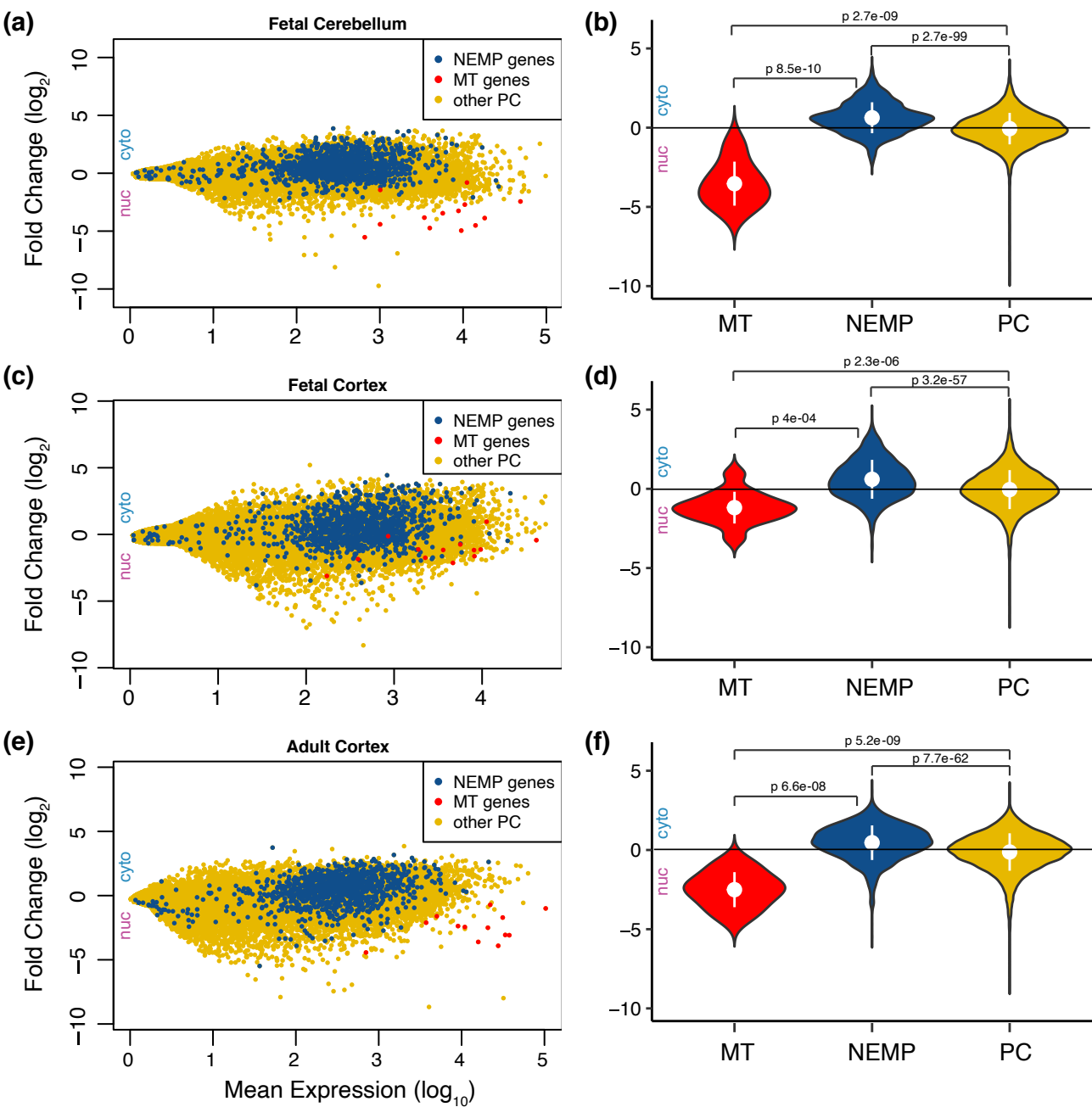

Supplement: Supplementary file 5 — Supplementary Figure S4. [file 41598_2021_83541_MOESM5_ESM.pdf]
