# Supplementary material for: Characterization of the nuclear and cytosolic transcriptomes in human brain tissue reveals new insights into the subcellular distribution of RNA transcripts
Source: Sci Rep. 2021 Feb 18;11:4076. doi: 10.1038/s41598-021-83541-1 (PMC7893067; doi:10.1038/s41598-021-83541-1)

Supplementary Figure 5
ENCODE DEGs were identified from longPolyA sequencing data for nine cell lines. NEMPs and protein-coding genes (PC) fold change distributions are shown by violin plots. Plot shows all genes (baseMean >=10 and Padj < 0.05).

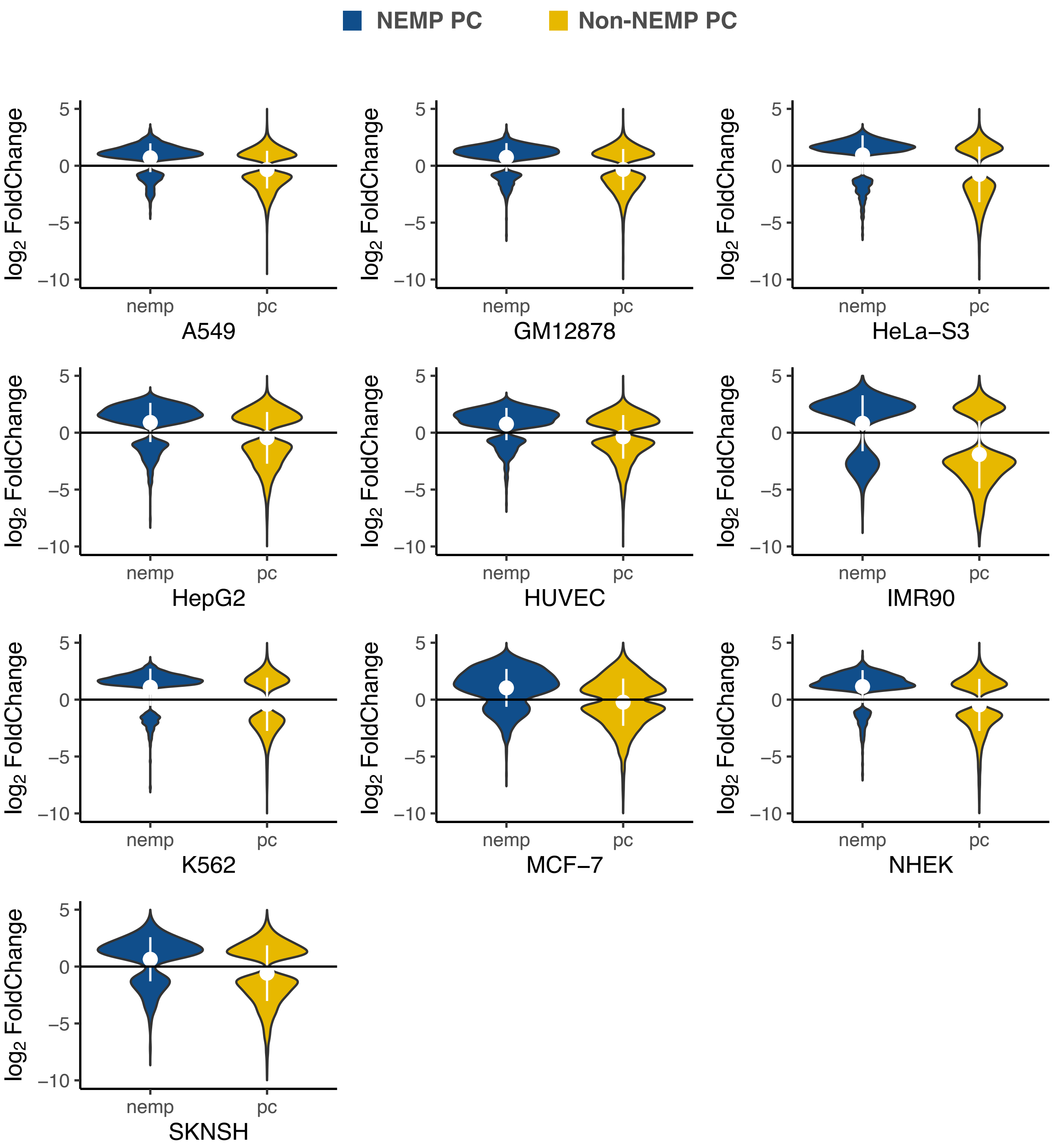

Supplement: Supplementary file 6 — Supplementary Figure S5. [file 41598_2021_83541_MOESM6_ESM.pdf]
