# Supplementary material for: Characterization of the nuclear and cytosolic transcriptomes in human brain tissue reveals new insights into the subcellular distribution of RNA transcripts
Source: Sci Rep. 2021 Feb 18;11:4076. doi: 10.1038/s41598-021-83541-1 (PMC7893067; doi:10.1038/s41598-021-83541-1)

Supplementary Figure 6

(A-C) MA plot of each brain tissue lncRNA genes upregulated in cytosol (blue) and upregulated in nucleus (magenta).

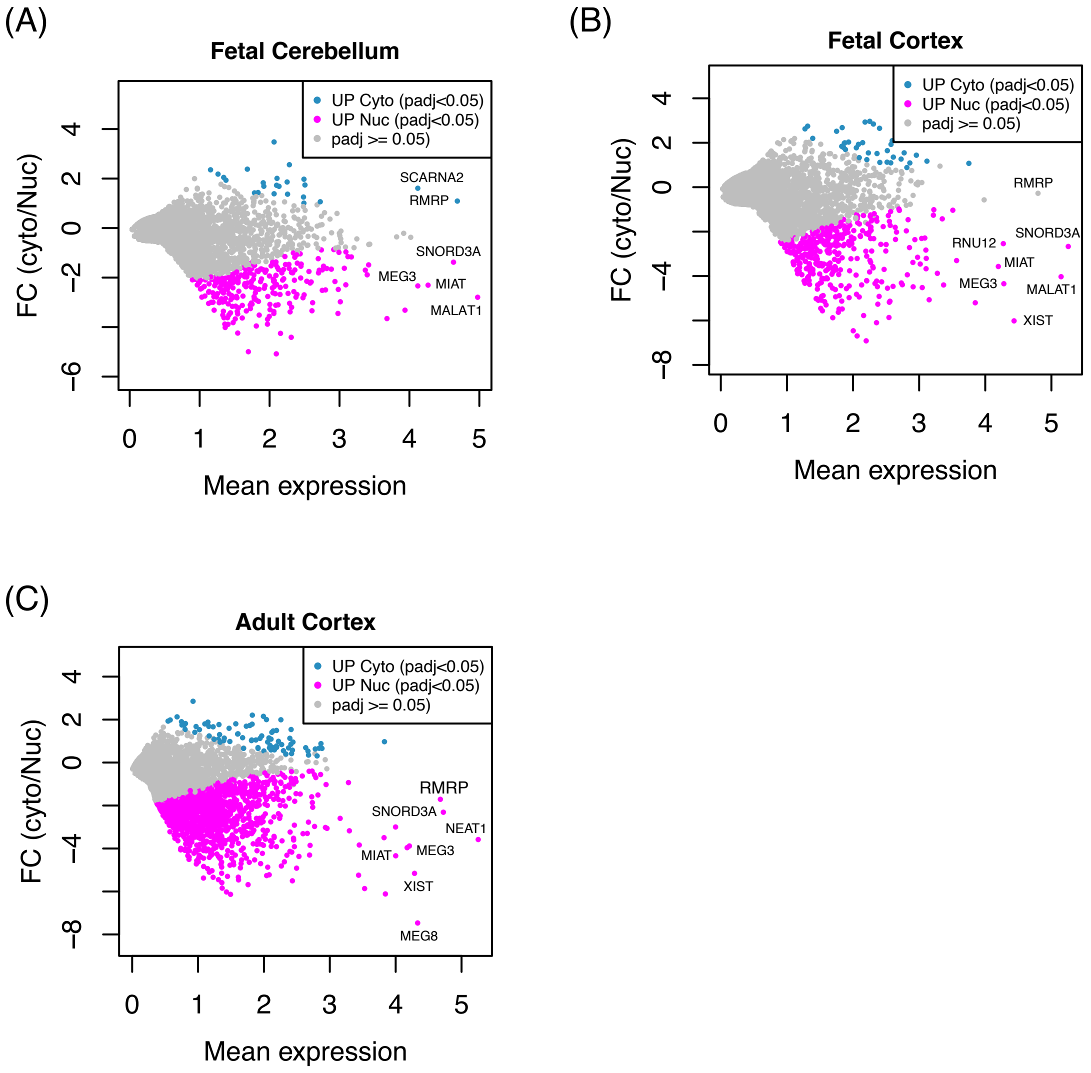

Supplement: Supplementary file 7 — Supplementary Figure S6. [file 41598_2021_83541_MOESM7_ESM.pdf]
