# Supplementary material for: Characterization of the nuclear and cytosolic transcriptomes in human brain tissue reveals new insights into the subcellular distribution of RNA transcripts
Source: Sci Rep. 2021 Feb 18;11:4076. doi: 10.1038/s41598-021-83541-1 (PMC7893067; doi:10.1038/s41598-021-83541-1)

Supplementary Figure 7

Overlap analysis between transcripts localized either in the cytosol or the nucleus between human and mouse.

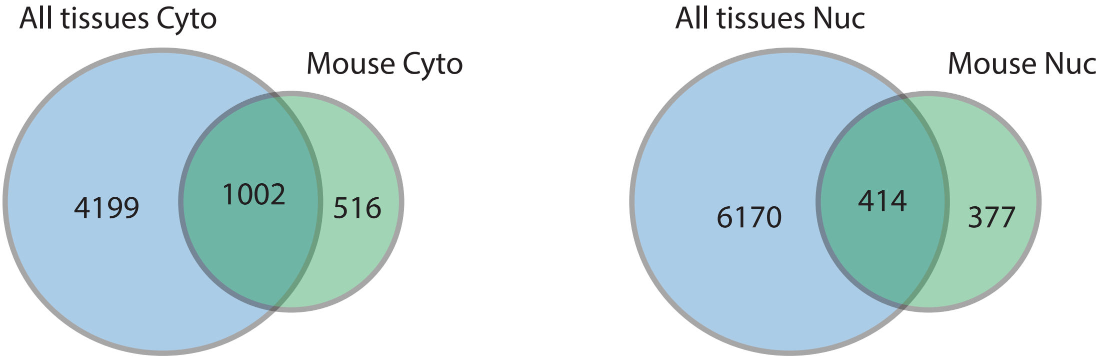

Supplement: Supplementary file 8 — Supplementary Figure S7. [file 41598_2021_83541_MOESM8_ESM.pdf]
