# Supplementary material for: Characterization of the nuclear and cytosolic transcriptomes in human brain tissue reveals new insights into the subcellular distribution of RNA transcripts
Source: Sci Rep. 2021 Feb 18;11:4076. doi: 10.1038/s41598-021-83541-1 (PMC7893067; doi:10.1038/s41598-021-83541-1)

Supplementary Figure 8

Bioanalyzer results for the purified RNA from the cytosol and the nucleus from on of the fetal frontal cortex samples. The ribosomal RNA only appeared in the cytosolic RNA fraction indicating as expected when nuclear RNA is free of cytoplasmic contamination.

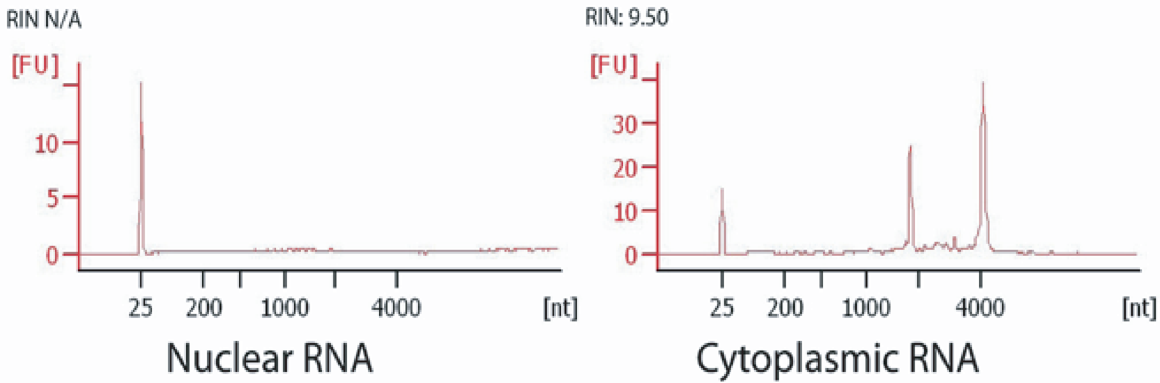

Supplement: Supplementary file 9 — Supplementary Figure S8. [file 41598_2021_83541_MOESM9_ESM.pdf]
