# Supplementary material for: Characterization of the nuclear and cytosolic transcriptomes in human brain tissue reveals new insights into the subcellular distribution of RNA transcripts
Source: Sci Rep. 2021 Feb 18;11:4076. doi: 10.1038/s41598-021-83541-1 (PMC7893067; doi:10.1038/s41598-021-83541-1)

Supplementary Figure 9

Expression means versus log2 fold changes (log2FC) for the adult cortex showing cytosolic and nuclear expression differences for protein-coding, lncRNA, snoRNA genes and snRNAs. Left panel shows where U6 falls on the MA-plot. MALAT, XIST and NEAT1 are also marked for comparison. The right panel shows the fold change distribution for genes in each of four biotypes, including snRNA (green).

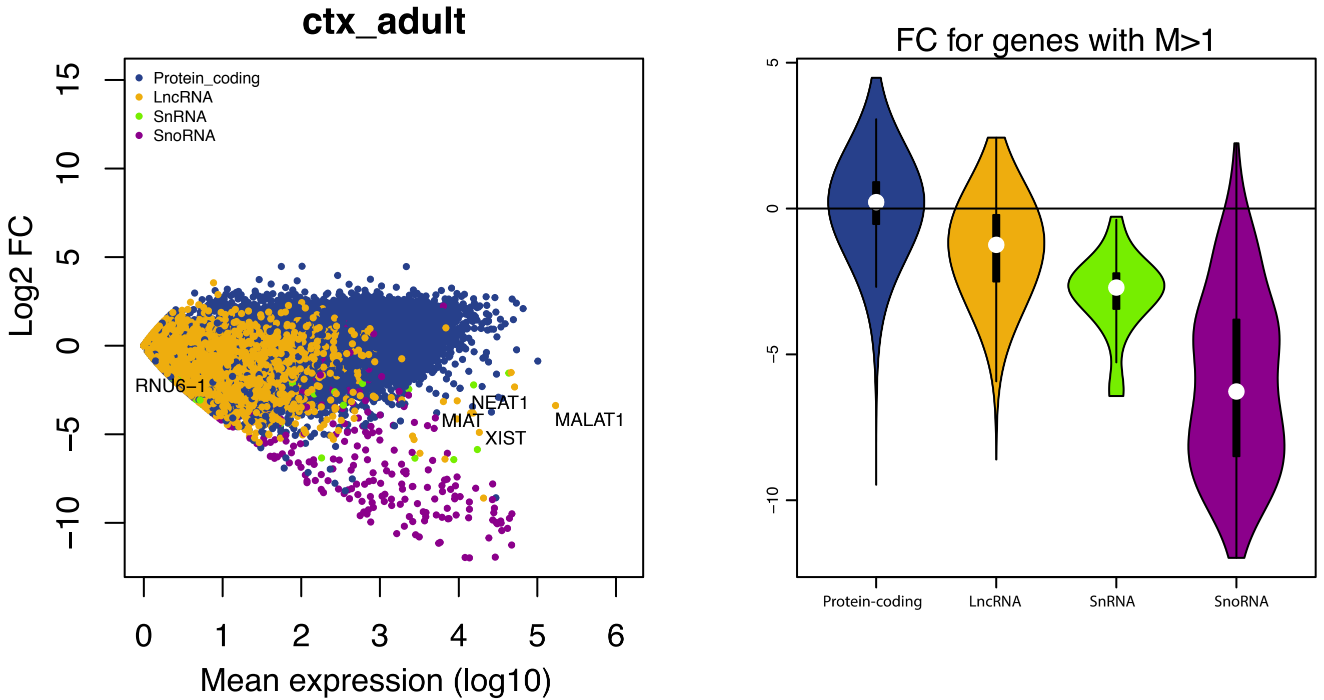

Supplement: Supplementary file 10 — Supplementary Figure S9. [file 41598_2021_83541_MOESM10_ESM.pdf]
